# A biomass map of the Brazilian Amazon from multisource remote sensing

**DOI:** 10.1038/s41597-023-02575-4

**Published:** 2023-09-30

**Authors:** Jean Pierre Ometto, Eric Bastos Gorgens, Francisca Rocha de Souza Pereira, Luciane Sato, Mauro Lúcio Rodrigures de Assis, Roberta Cantinho, Marcos Longo, Aline Daniele Jacon, Michael Keller

**Affiliations:** 1 Instituto Nacional de Pesquisas Espaciais (INPE), Av dos Astronautas, 1758, 12227-010 São José dos Campos, SP Brazil; 2 Universidade Federal dos Vales do Jequitinhonha e Mucuri. Campus JK. Rodovia MGT 367 – Km 583, n° 5000, Alto da Jacuba, 39100-000 Diamantina, MG, Brazil; 3 Centro de Desenvolvimento Sustentável, Universidade de Brasília, 70910-900 Brasília, DF Brazil; 4 Climate and Ecosystem Sciences Division, Lawrence Berkeley National Laboratory, 1 Cyclotron Rd, Berkeley, CA 94720 USA; 5 USDA Forest Service, International Institute of Tropical Forestry, Rio Piedras, Puerto Rico USA & Jet Propulsion Laboratory, Pasadena, CA 91011 USA

**Keywords:** Environmental impact, Forestry

## Abstract

The Amazon Forest, the largest contiguous tropical forest in the world, stores a significant fraction of the carbon on land. Changes in climate and land use affect total carbon stocks, making it critical to continuously update and revise the best estimates for the region, particularly considering changes in forest dynamics. Forest inventory data cover only a tiny fraction of the Amazon region, and the coverage is not sufficient to ensure reliable data interpolation and validation. This paper presents a new forest above-ground biomass map for the Brazilian Amazon and the associated uncertainty both with a resolution of 250 meters and baseline for the satellite dataset the year of 2016 (i.e., the year of the satellite observation). A significant increase in data availability from forest inventories and remote sensing has enabled progress towards high-resolution biomass estimates. This work uses the largest airborne LiDAR database ever collected in the Amazon, mapping 360,000 km^2^ through transects distributed in all vegetation categories in the region. The map uses airborne laser scanning (ALS) data calibrated by field forest inventories that are extrapolated to the region using a machine learning approach with inputs from Synthetic Aperture Radar (PALSAR), vegetation indices obtained from the Moderate-Resolution Imaging Spectroradiometer (MODIS) satellite, and precipitation information from the Tropical Rainfall Measuring Mission (TRMM). A total of 174 field inventories geolocated using a Differential Global Positioning System (DGPS) were used to validate the biomass estimations. The experimental design allowed for a comprehensive representation of several vegetation types, producing an above-ground biomass map varying from a maximum value of 518 Mg ha^−1^, a mean of 174 Mg ha^−1^, and a standard deviation of 102 Mg ha^−1^. This unique dataset enabled a better representation of the regional distribution of the forest biomass and structure, providing further studies and critical information for decision-making concerning forest conservation, planning, carbon emissions estimate, and mechanisms for supporting carbon emissions reductions.

## Background & Summary

The Amazon basin in South America is an emblematic region in the global environmental discussion due to its extensive ecosystems^1,2^, its diverse biodiversity^1,3,4^, its climate regulation and forcing^1,5,6^, and its benefits to people^4,7^. The South American Amazon forests contain between 95 and 200 Pg of carbon stored in living biomass^8–10^. Until recently, the Amazon Forest acted as a net sink of carbon, an important ecosystem service to the planet^3,11^. However, recent findings suggest that the forest may be losing its sink capacity and becoming a carbon source to the atmosphere^12^.

The science of the global carbon (C) cycle has continuously evolved, leading to important advances in estimating the fossil fuel component of the cycle. This highlights the urgency of addressing the critical and constant increase in greenhouse gas concentrations in the atmosphere^6,13^. The non-fossil fuel portion of the global C cycle carries great uncertainty. It is associated with land-based activities, such as changes in vegetation cover (especially in the tropics) or agriculture (where CO_2_ emissions are less critical than CH_4_ or N_2_O)^14^.

Carbon stored on land is equivalent to decades of fossil fuel emissions, and increasing anthropogenic disturbance reduces the carbon residence time in natural ecosystems^15^. Forests also play a role in many ecological processes and dynamics critical to humanity, such as photosynthesis, the hydrological cycle, and energy flow^7,16,17^. Estimates of carbon stocks and fluxes for the Amazon region are highly uncertain. Comparisons of the published maps reveal substantial differences in forest biomass from this region, which can lead to high uncertainty when calculating carbon emissions from deforestation, forest degradation, and other changes in land cover^18–20^.

The forest ecosystem is complex and often difficult to access. Ground observations on most tropical forests have limited temporal and spatial distribution, typically covering an area of less than 1 hectare, and their spatial distribution may not be representative of landscapes^21^. Furthermore, their limited size may induce significant uncertainties in biomass estimates due to the presence or absence of large individual trees^22,23^. Several studies have been conducted to better estimate and analyze the spatial distribution of the forest biomass across the Amazon^23–25^.

Deforestation and associated land-use change in the Amazon are heterogeneous and patchy, leading to uncertainty in estimates of carbon emissions unless spatial variability is captured. Well-calibrated biomass maps can provide the information needed to reduce this uncertainty. Biomass maps present continuous spatial distribution values of forest biomass density, covering the whole Amazon where ground data is limited^26^. The map historically adopted by the Brazilian National Communication to the United Nations Framework Convention on Climate Change (UNFCCC) to estimate the spatial variation of above- and below-ground biomass in the Brazilian Amazon is based on remote sensing and interpolation of large-scale forest inventories conducted from the early 1970s to the early 1980s, with additional field measurements added later. In its last submission, Brazil incorporated the results of this study as a parameter for calculating emissions from the Land Use, Land-Use Change, and Forestry (LULUCF) sector.

We present a new biomass map for the Brazilian Amazon as a reference for the scientific community and government. The map was produced using the largest Light Detection and Ranging (LiDAR) database collected from aircraft flying over the Brazilian Amazon region. This map can be used to support further work and discussions about carbon fluxes in the tropical forest of the Amazon, project future atmospheric CO_2_ concentrations, and determine mitigation policies. Possible applications include contributing to UNFCCC reports, the Intergovernmental Panel on Climate Change (IPCC), and incentivizing reductions in greenhouse gas emissions from deforestation and forest degradation (REDD+). Additionally, the map and the original dataset can support and help models estimate carbon losses and gains due to human activities and climate change.

## Methods

### Airborne laser scanning collection

In two consecutives campaigns (2016/2017 and 2017/2018), we collected 901 LiDAR transects across the Brazilian Amazon. From those, 613 of these transects were randomly distributed over the forest and secondary forest, 133 were randomly distributed over the deforestation arch, and 155 overlapped field plots to allow for model calibration. Each transect covered a minimum of 375 hectares (12.5 km × 300 m) and was surveyed by emitting full-waveform laser pulses from a Trimble Harrier 68i airborne sensor (Trimble; Sunnyvale, CA) aboard a Cessna aircraft (model 206). The average point density was set at four returns per m² considering all returns, the field of view was 30°, the flying altitude was 600 m, and the transect width on the ground was approximately 494 m. Global Navigation Satellite System (GNSS) data were collected on a dual-frequency receiver (L1/L2). The pulse footprint was below 30 cm, based on a divergence angle between 0.1 and 0.3 milliradians. Horizontal and vertical accuracy were controlled using ground control station during the flights campaign and was specified to be under 1 m and 0.5 m, respectively. The contractor had the responsibility to deliver the products attending the required accuracy.

The discrete airborne cloud about each LiDAR transects are hosted at Zenodo repository^27–30^. The LiDAR point clouds are separated by states in the Brazilian Amazon.

In 2016/2017, we sampled transects randomly distributed across the PRODES-INPE forest mask (PRODES, 2015) and secondary vegetation (forest regrown after complete forest clearing) from INPE-TerraClass mask (TerraClass, 2014). To calibrate and validate the airborne LiDAR predictions of biomass, we intentionally overlapped some transects with field plots from a number of research partners. In 2017/2018, we expanded the number of transects to improving the representation of secondary forest based on INPE-TerraClass mask (TerraClass, 2014). The metadata about each transect is included in the shapefile hosted at Zenodo repository^31^. Some of the information available for each transect includes ID (unique identification), year of acquisition, presence of field data, random positioning in the mask, EPSG, and other relevant details.

To position the transects, we randomly generated center points with X, Y coordinates and azimuth. We visually inspected the start points to ensure they were within the forest or secondary vegetation mask. If the start point was not within a forest, as seen by satellite image, we discarded the seed point and selected another one. For each point, we created a shapefile with a 12.5 km × 300 m polygon. For both campaigns, if there were any conflicts with the flight plan (e.g., proximity to an airport or military restrictions), the company making the flights requested repositioning it to the closest allowed area.

### Biomass map computation

**Fig. 1:**
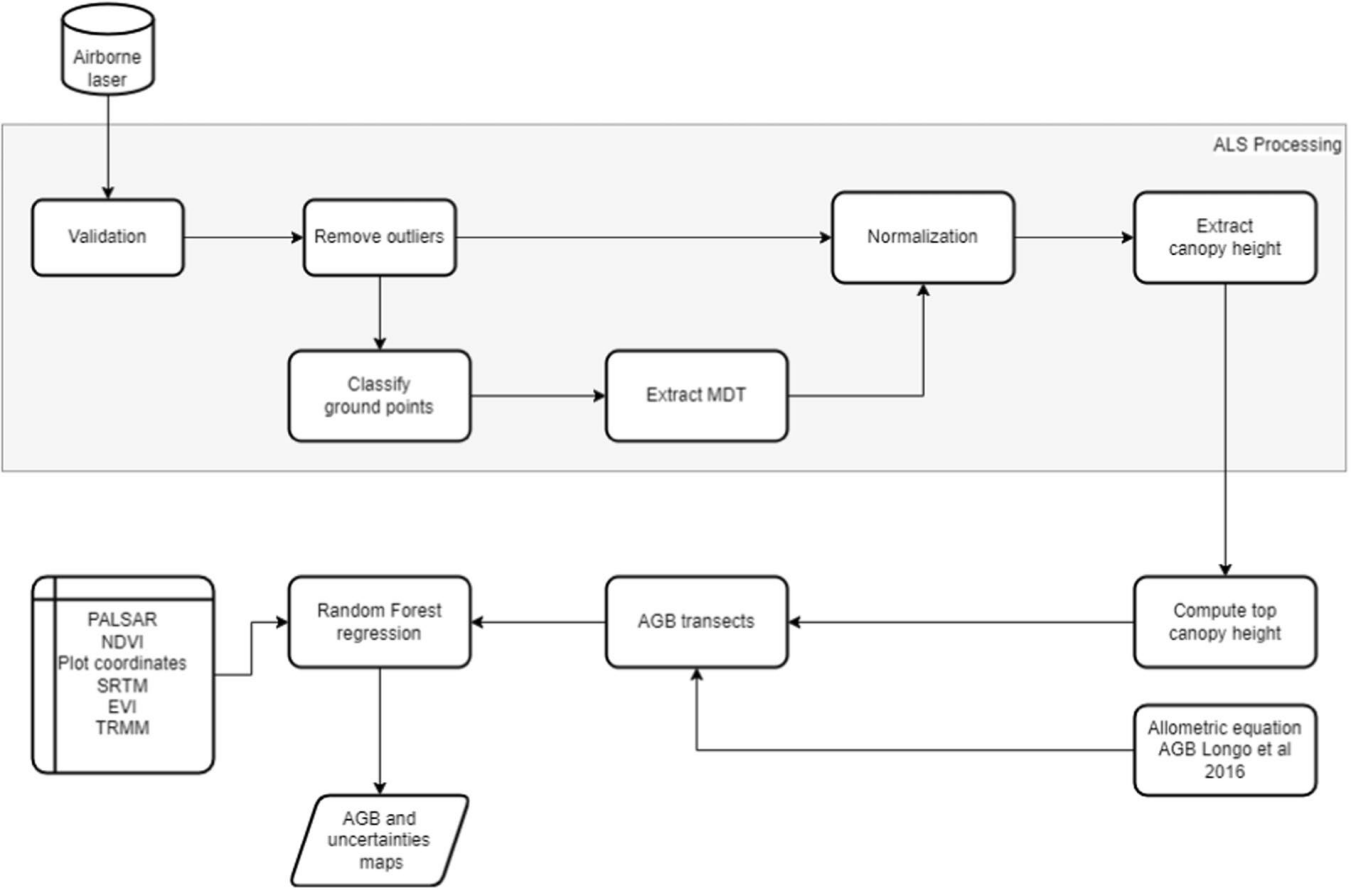
Diagram presenting the processing steps for biomass map creation.

The above-ground biomass (AGB) map was computed in parallel using the Anaconda environment and H2O data modeling library (http://docs.h2o.ai/) on a Windows Server. The biomass was estimated from LiDAR top canopy height metric based on a calibration using field survey plots across the region^32,33^. The training dataset and layers for biomass prediction is hosted at Zenodo repository^34^. Machine learning was used to calibrate a regional biomass map based on radar and passive optical satellite data. The uncertainty of the different levels of the data was also analyzed to produce a biomass estimation uncertainty map (Fig. 1).

The top canopy height metric processing steps from the LiDAR transects are summarized as follows: (1) removing outliers from the point clouds, (2) classifying the ground points, (3) building a digital terrain model (DTM) at 1 m resolution, (4) normalizing the point cloud and, (5) creating the canopy height model (CHM) with the 1-m resolution. Each canopy height model were processed, generating top canopy height by aggregating the 1-meter CHM pixels for 50 m cells by average^32^. All the computation was performed in LasTools and PostGIS-Spatial PostgreSQL platform.

Returns recorded by the LiDAR equipment that are not related to the forest canopy or the ground are referred to as outliers. Common outliers are produced by the laser hitting birds, water vapor clouds above the forest, and sensor errors. To detect and exclude isolated points, we implemented an algorithm to find points surrounded by a few other points in their 4 by 4 by 4 voxels of 1 meter in size. The threshold for being considered an isolated point was set to 5 neighbors.

The digital terrain model (DTM) was created based on the returns classified as ground by the terrain curvature filter^35^. This algorithm creates a sparse triangulated irregular network (TIN) from the neighborhood minima. It progressively densifies the TIN, adding new points to the TIN if the points are below a threshold. The parameters used to compute the threshold are the standard values in Lastools for the angle between the point and the TIN facet, and the closest facet nodes’ distance. The produced DTM was then used to normalize the point cloud. Each return from the original cloud had its elevation subtracted by the elevation in the corresponding DTM pixel. The output was a normalized cloud, where the z coordinates of each return indicated the height from the ground.

The canopy height model (CHM) was computed by assigning the highest return value within each 1 × 1 m grid cell to the grid cell. The CHM was rescaled to a 50 m resolution grid (TCH 50) for calibration with filed data and was rescaled by averaging the elevation of the corresponding pixels, producing the top canopy height (TCH)^28^. The forest biomass (kg m^−2^), derived from LiDAR data, was calculated using TCH as predictor^32^.

$${\rm{\$\$AGB}}={\rm{2}}\ast (0.025\ast {{\rm{TCH}}}^{\wedge }{\rm{\{1}}{\rm{.99\}}}){\rm{\$\$}}$$

To generate a wall-to-wall map of the Brazilian Amazon we rescaled the LiDAR data from 50 m to 250 m resolution, which is the same resolution as the satellite data. The Brazilian Amazon were represented by 68,629,072 250 m pixels, and 141,032 pixels have LiDAR information and therefore had the AGB estimated and converted to Mg ha^−1^. We trained a Random Forest (RF) model^36^ using the AGB estimated pixels and remote sensing layers formed by: MODIS vegetation indices, Shuttle Radar Topography Mission (SRTM) data, Tropical Rainfall Measuring Mission (TRMM), and Phased Array type L-band Synthetic Aperture Radar 2 (PALSAR-2) data, along with the central coordinates of each 250 m pixel. Derived from MODIS, we used the Vegetation Indices 16-Day L3 Global 250 m temporal series (MOD13Q1) products from 2016, including the Normalized Difference Vegetation Index (NDVI) and Enhanced Vegetation Index (EVI), from MODIS. From TRMM we used the 3B43 V6 precipitation data, with each pixel value representing the monthly accumulated precipitation from 1998 to 2016 at a resolution of 0.25 degrees. From PALSAR-2, provided as Gamma-0 backscatter, we used the L-band image in the HH and HV polarizations, acquired in 2016. When necessary, the remote sensing products were resampled by mean to a 250 m grid.

Random Forest models were tested using the H2O_Flow platform and produced the best model based on RMSE and R², containing NDVI q3 (q3 refer to the third quartile), PALSAR-2 HV, TRMM mean, X, SRTM, Y, PALSAR-2 HH, EVI q1, EVI mean, NDVI mean, NDVI q1 (q1 refer to the first quartile), EVI q3 (ordered by importance; see Table 1). The RF model used to extrapolate the prediction and generate the AGB map for the entire Amazon biome presented an R² = 0.75 and RMSE = 27 Mg ha^−1^ (15,5%). The variable importance was determined by calculating each variable’s relative influence, which reduced the squared error as the difference in squared error between a node and its children’s nodes. The squared error for each node was the reduction in the variance of the response value within that node.

**Table 1:** Variable importance after RMSE stabilization during RF training.

Variable	Scaled importance	Importance as percentage
NDVI q3	1	0.2005
PALSAR-2 HV	0.7704	0.1544
TRMM mean	0.6908	0.1385
X	0.6065	0.1216
SRTM	0.5159	0.1034
Y	0.4857	0.0974
PALSAR-2 HH	0.4416	0.0885
EVI q1	0.1546	0.0310
EVI mean	0.1098	0.0220
NDVI mean	0.0816	0.0164
NDVI q1	0.0685	0.0137
EVI q3	0.0632	0.0127

The uncertainty associated with the AGB map was calculated by propagating the uncertainties through the different levels of biomass estimation: field plots (first level), LiDAR transects (second level), and satellite layers (third level)^32^. The first and second levels were based on Longo *et al*. 2016 model and took into account: (1) the uncertainty associated with AGB estimates from the forest inventory plots used to calibrate the LiDAR model, (2) the uncertainty related to the limited sampling of forest inventory plots overlapped with the LiDAR surveys; and (3) the uncertainty related to the fraction of variance that cannot be captured by the fitted model^28^. The uncertainty was estimated first for each 50-m cell derived from LiDAR data and then, then resampled to 250 m to match the wall-to-wall map.

The third level of uncertainty (related to the satellite layers) was propagated to the wall-to-wall map in two steps: (1) a normal distribution (mean and standard deviation) of the AGB was simulated using the total uncertainty and AGB value of each cell (spatial resolution of 250 m) of the transect; (2) one thousand AGB maps were generated using the normal distribution values for AGB, remote sensing variables, and random forest regression model, in the same way the final AGB map was modeled. The wall-to-wall uncertainty map was generated by calculating the standard deviation of AGB of each cell. This process allowed us to obtain uncertainty estimates for each pixel of the final AGB map. The simulation process was implemented in four Linux virtual machines with 256 Mb memory and 64 processors each, taking 40 hours to generate the uncertainty map.

## Data Records

### LiDAR transects

**Fig. 2:**
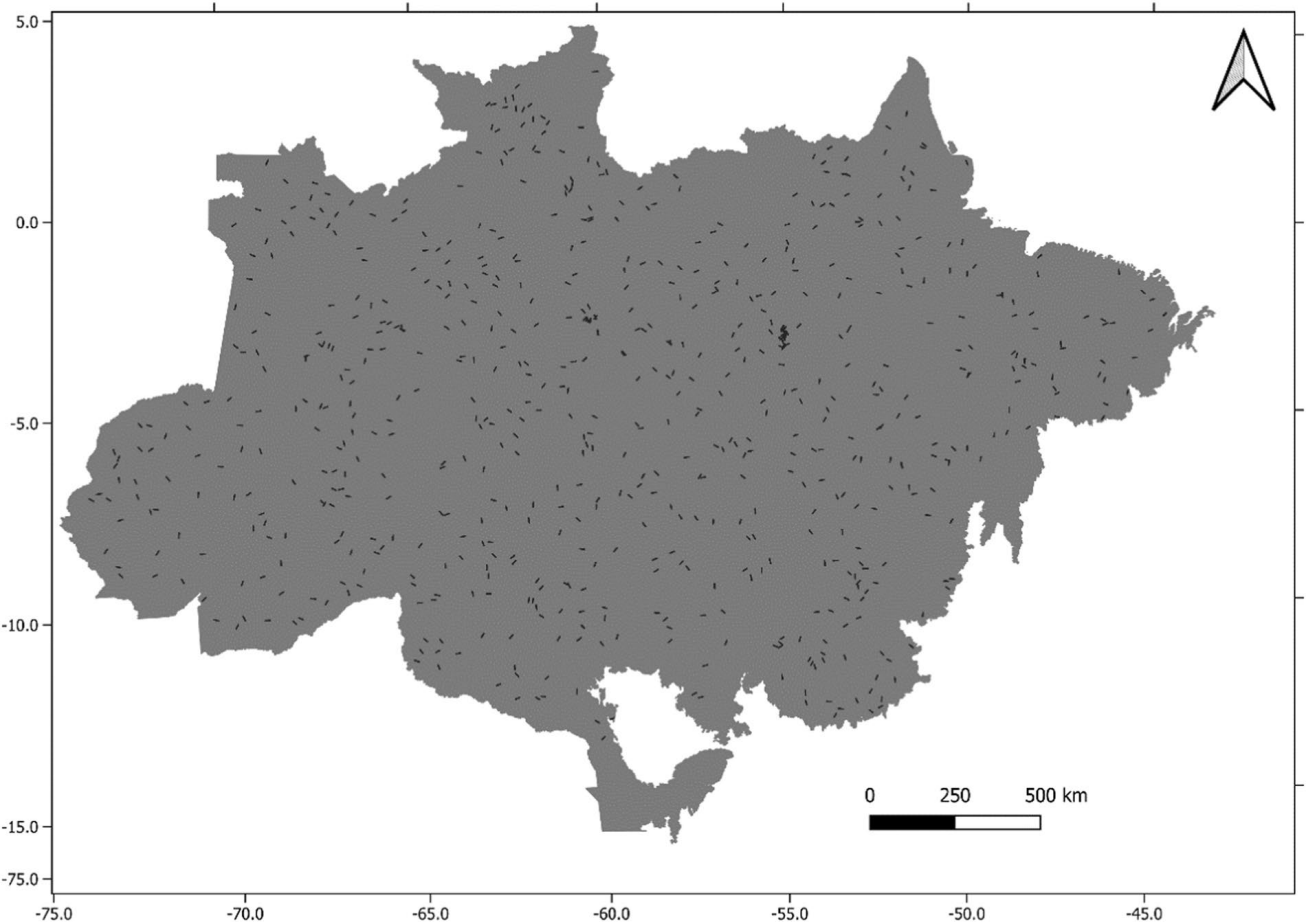
Location of LiDAR transects (black) location over the Amazon biome (dark gray).

The transects boundaries, location and attributes are summarized in a shapefile format, deposited in the Zenodo^31^ (Fig. 2). Each transect is stored in a single LAZ file, with a unique name matching the shapefile. The metrics were extracted from the original point cloud, including basic outlier cleaning.

### Biomass map

**Fig. 3:**
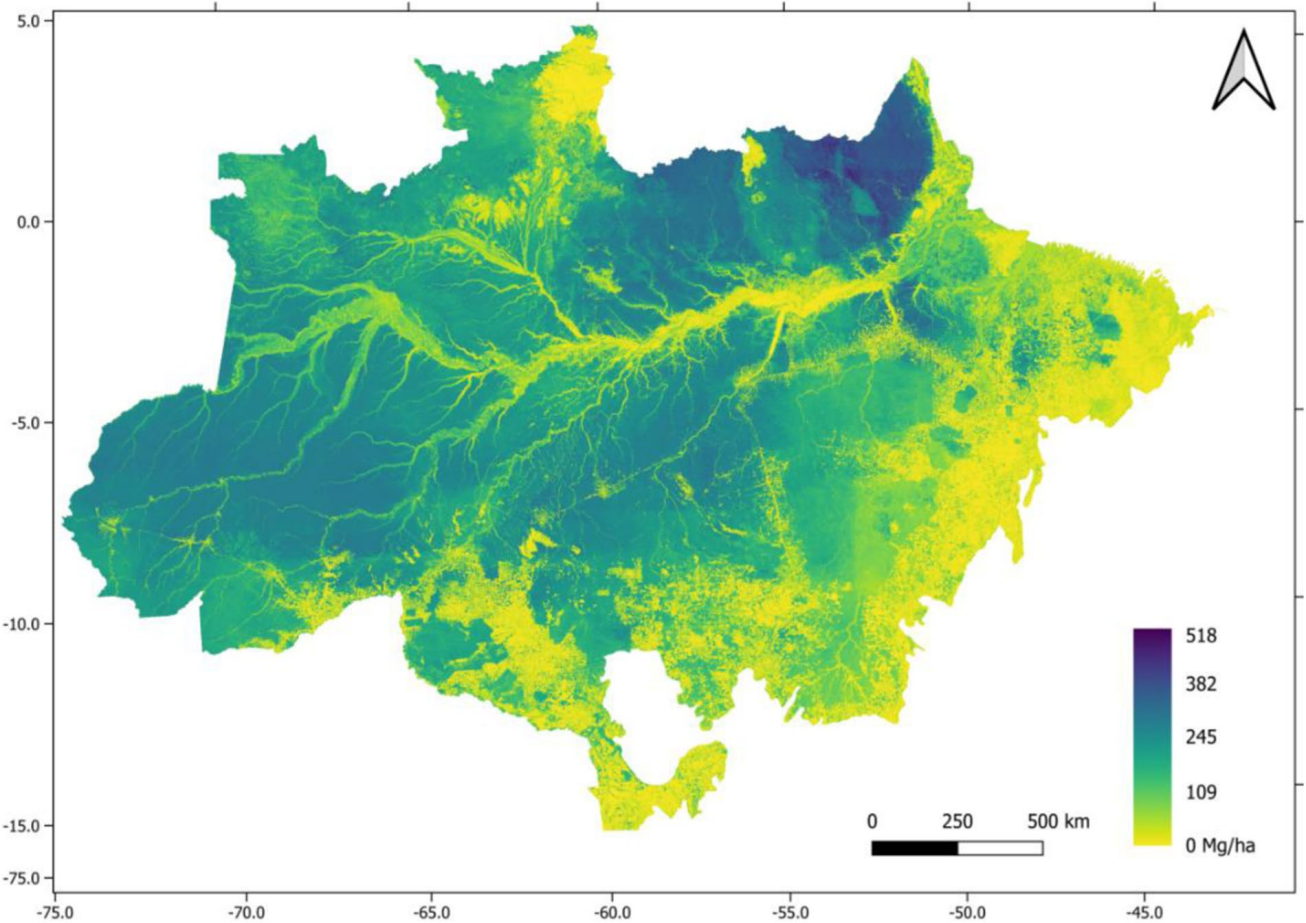
Distribution of Above ground biomass map (Mg ha^−1^) across de Amazon biome. Note the relatively lower biomass along rivers and streams linked to riparian vegetation.

The AGB final map (further referred as EBA - Estimativa de Biomassa para a Amazônia - map) presented a maximum AGB value of 518 Mg ha^−1^, a mean AGB of 174 Mg ha^−1^, and a standard deviation of 102 Mg ha^−1^. The map is provided in TIF format, represented using EPSG 4326 (Fig. 3). The biomass map is deposited in the Zenodo repository^37^.

### Biomass uncertainty map

**Fig. 4:**
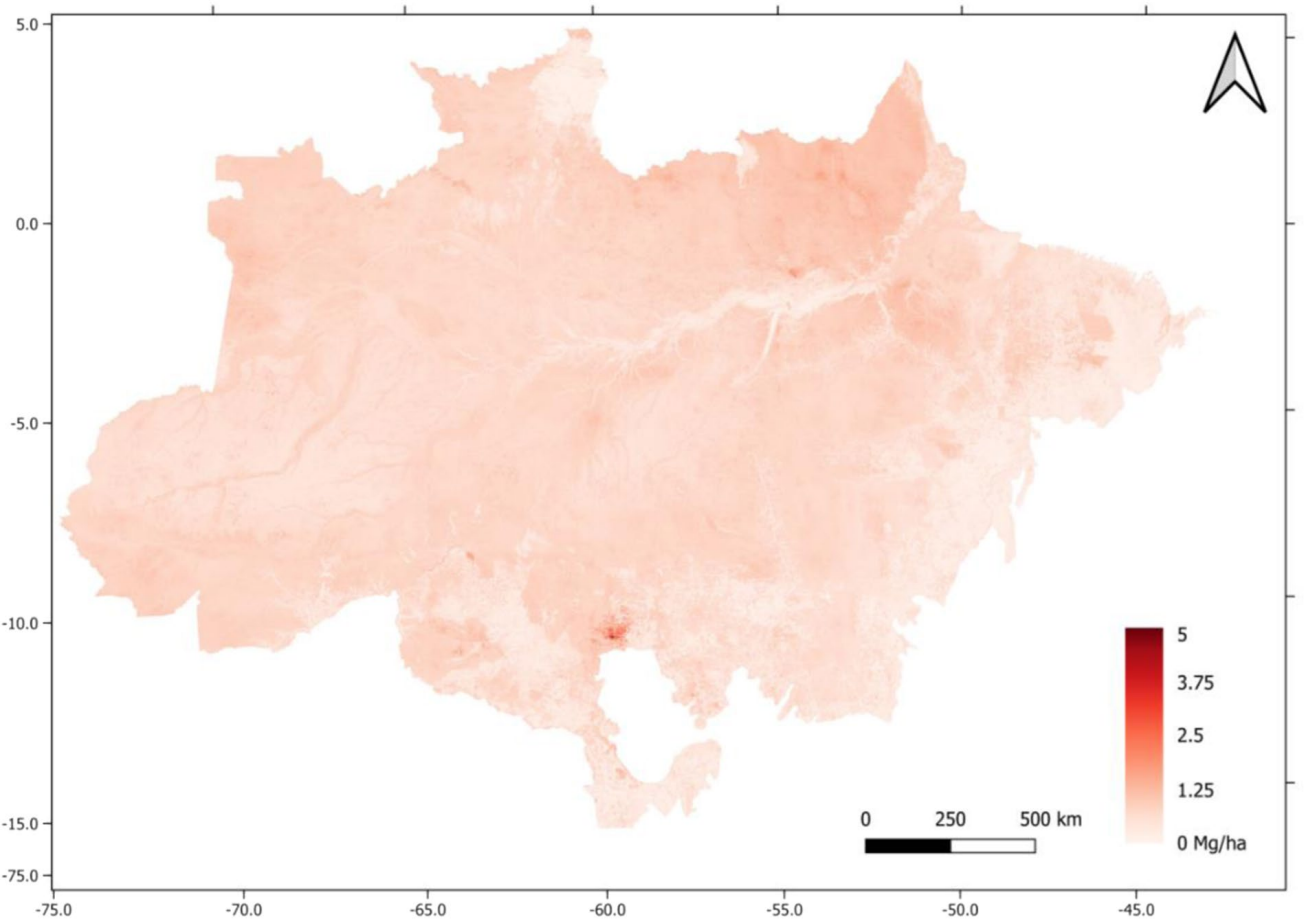
Uncertainty of aboveground biomass estimates (Mg ha^−1^) across the Amazon biome, map for biomass estimations provided in Mg ha^−1^.

The uncertainty map is provided in TIF format, projected using EPSG 4326. The information is offered in Mg ha^−1^ (Fig. 4). The biomass map is deposited in the Zenodo repository^37^.

## Technical Validation

The distribution of transects captured the most representative vegetation types in the Amazon. The Open (in Brazilian classification: Floresta Ombrófila Aberta) and Dense Forests (in Brazilian classification: Floresta Ombrófila Densa), representing 27.23% and 54.29% of the Amazon received 30.82% and 49.08% of the transects. The seasonal forest (in Brazilian classification: Floresta Estacional Semidecidual) (5,16%) and open savannah (in Brazilian classification: Campinarana, 5.08%) received, respectively 7.46% and 5.17% of all transects (Table 2). The number of transects flown in different Brazilian Amazonia states (in alphabetical order) are as follow: (20) Acre; (25) Amapá; (286) Amazonas; (24) Maranhão; (127) Mato Grosso; (301) Pará; (71) Rondônia; (46) Roraima; (1) Tocantins.

**Table 2:** The distribution of transects captured the most representative vegetation types in the Amazon region.

Vegetation Type	Amazon Region	Percent of transects
Floresta Ombrófila Aberta	27.23%	30,82%
Floresta Ombrófila Densa	54.29%	49.08%
Floresta Estacional Semidecidual	5.16%	7.46%
Campinarana	5.08%	5.17%

A total of 156 field plots were used to validate the Above-Ground Biomass (AGB) estimated by the LiDAR-based model. The field biomass was computed through inventories of trees, palms, and lianas’ biomass within each plot. The estimated AGB of the plot (kg m^−2^) was obtained by dividing the total biomass of individuals within the plot (kg) by the area of the plot (m²). The individual AGB allometric equation for live trees was from^38^, for live palms from^39^, and for live lianas from^40^. The data was processed and delivered by the research partners. The models were constructed to estimate AGB kg, based on diameter of breast height (1.3 m - DBH) in cm, total height (Ht) in meters; and wood density in g cm^−3^ (ρ). The wood density value was established by tree species, genera, or family, based on^41^.

**Fig. 5:**
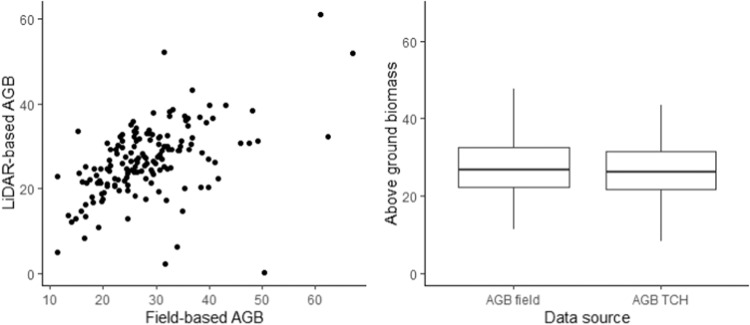
Above ground estimated by the model (kg m^−2^).

To validate the AGB estimated from LiDAR-based model, we cropped the LiDAR point cloud to the same extent of each field plot and estimated the above ground biomass. All the field plots were geo-located using the Differential Global Positioning System (DGPS), allowing accurate correspondence to the LiDAR point cloud data. The field AGB and LiDAR AGB have statistically similar mean values (~28 kg m^−2^) (Fig. 5). The Wilcoxon-Mann-Whitney test indicates that field AGB and LiDAR AGB are statistically similar (Wilcoxon rank sum test data W = 11141, p-value = 0.5917).

This effort aims to provide the largest database on LiDAR information of the Amazon Forest and a biomass map to inform decision makers (e.g., National commitments, funding mechanisms such as REDD+, and forest conservation strategies) with the most accurate information regarding the carbon content of the above ground vegetation in this region. Considering the implications of this information to several initiatives, we compared the map presented here Amazon with the biomass map present in the 3rd Brazilian National Communication map to the UNFCCC (United Nations Framework Convention on Climate Change).

**Fig. 6:**
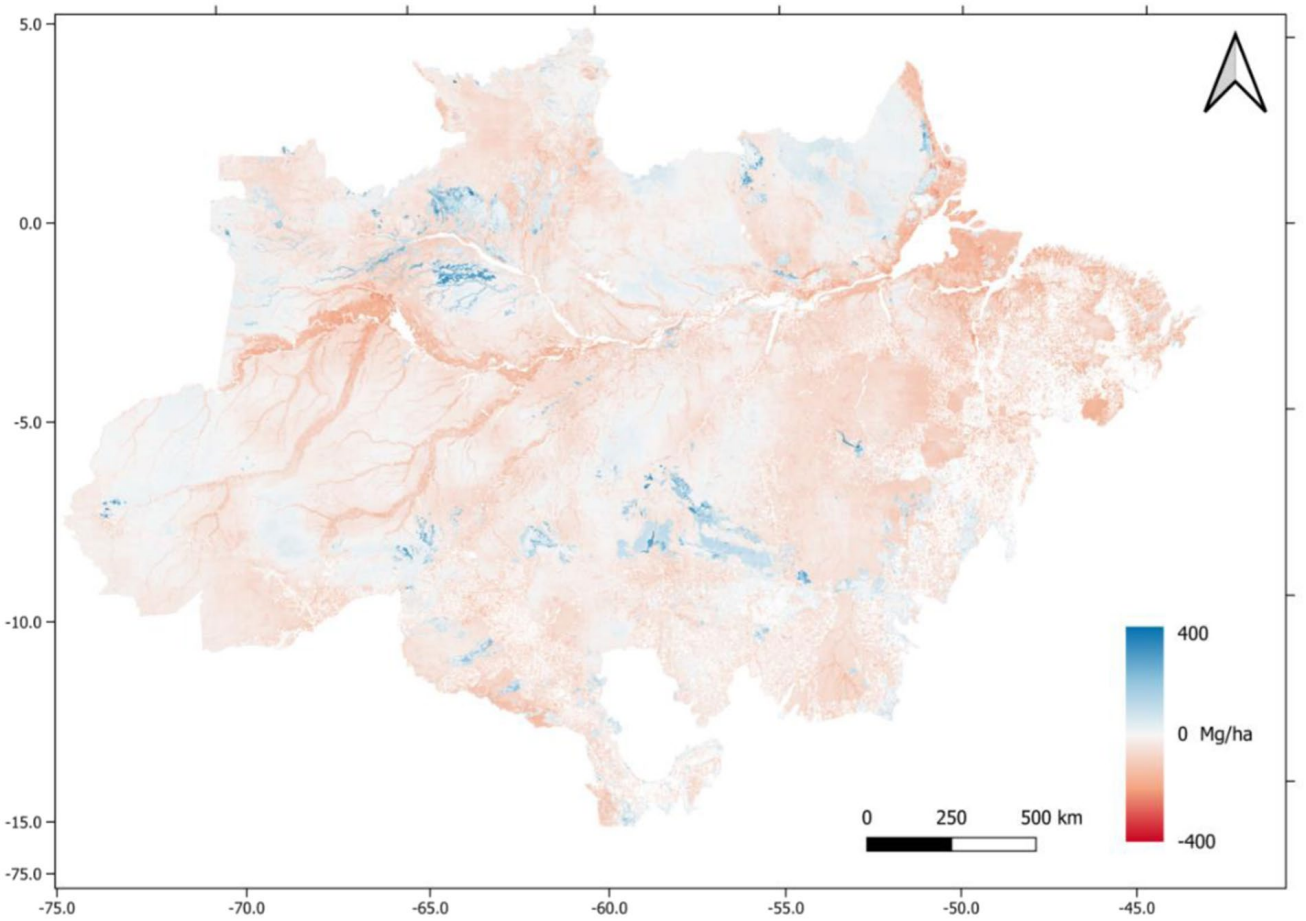
Comparison with the 3rd Brazilian National Communication. Negative values (red) indicate lower values to 3rd National Communication. Positive values (blue) indicate higher values to the new estimations.

Our AGB map was converted to carbon (AGBC) by multiplying it by a factor of 0.47^42^ to enable comparison with the 3rd Brazilian National Communication^43^. The 3rd Brazilian National Communication (3NC) was resampled by the nearest neighbor method to match the same resolution. To prevent bias, only non-anthropized regions were compared (based on PRODES-INPE mask) (Fig. 6).

The AGBC values in our map were generally lower than those reported in the 3NC from Brazil. This was especially true in riparian zones, the western portion of the basin, and areas affected by human influence. The 3rd National Communication estimated biomass using 1 × 1 km cells, and the carbon stock was regionally estimated by interpolating the basal area of *RadamBrasil* field plots and vegetation classes related to the sampling grid. In areas where *RadamBrasil* data was not collected, a single biomass value was assigned to the dominant vegetation class. It is worth noting that the biomass map of the 3NC showed an important evolution in statistics in the spatial interpolation of basal area data and the revisiting of allometric equations compared to the estimation presented in previous Brazilian National Communications.

**Fig. 7:**
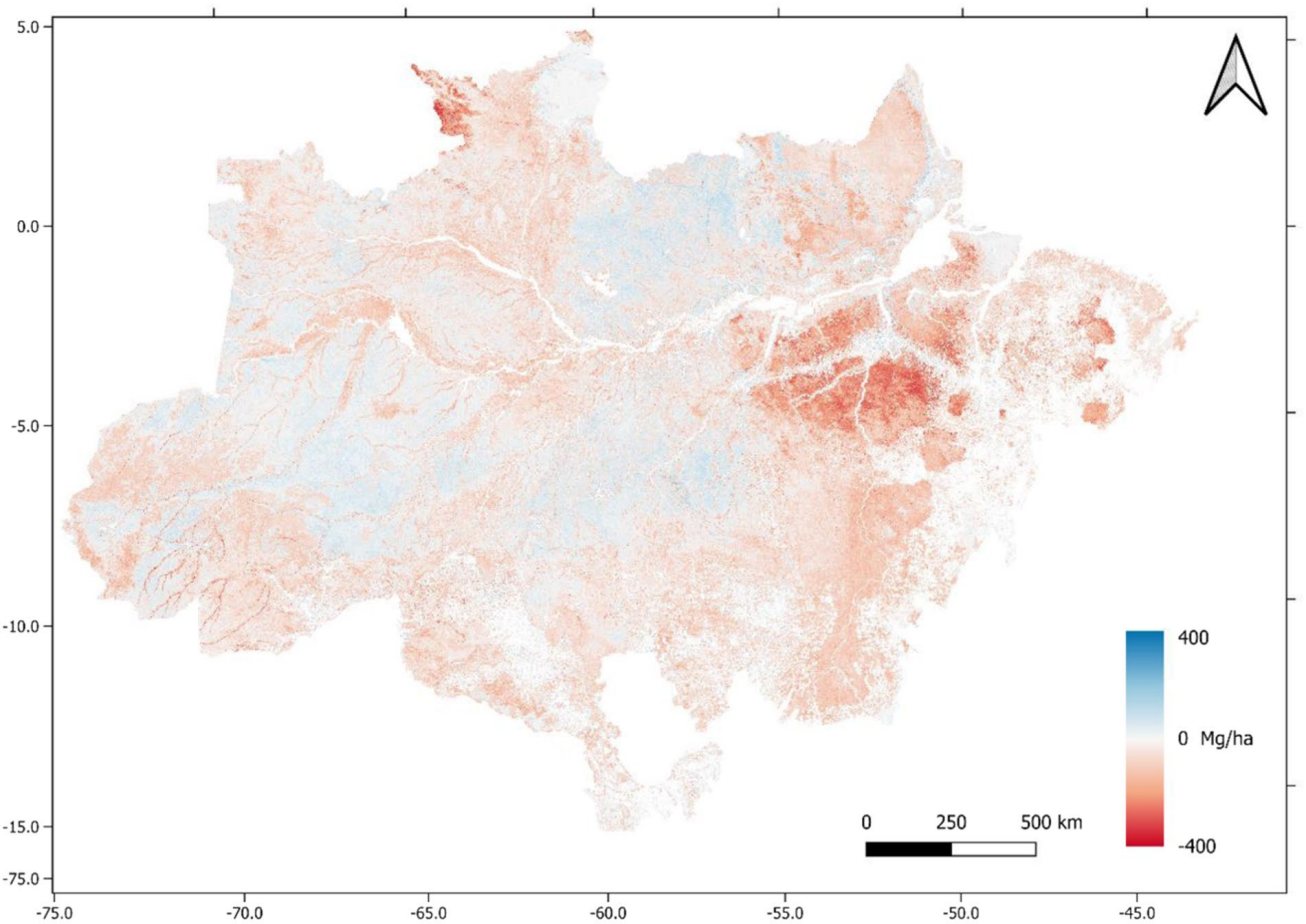
Comparison with the Avitabile *et al*.^23^ map. Negative values (red) indicate lower values to Avitabile *et al*.^23^ map. Positive values (blue) indicate higher values to the new estimations. .

A comparison was also performed between our biomass map with a pan-tropical biomass map produced by Avitabile *et al*.^23^. The map was resampled from 50 m by the nearest neighbor method to match the resolution of 250 m. To prevent bias, only non-anthropized regions were compared (Fig. 7). Comparing the two maps, the greatest differences in negative values occurred in the central region of the state of Pará and in the western region of the state of Roraima.

The comparison between the maps sought to present a relative consistency in the estimates. Notably, the most considerable differences for less are linked to areas close to rivers (i.e., riparian vegetation), which may be related to the lack of LiDAR points in flooded soils. The data used to generate the 3rd National Communication map came from RADAM, whose inventory data were from 1970. According to some studies (GATTI *et al*.^12^), some regions of the Amazon forest are losing biomass due to the indirect effects of deforestation, which could be reflected in a more up-to-date data collection, such as the LiDAR of this study (2016/2017). We need to explore this variation in future studies better. Nonetheless, the spatial distribution of the transects allowed a better characterization of the vertical structure of the forest. These data are absent in the third communication (2019) and Avitable *et al*.^23^ maps.

## Usage Notes

Net carbon losses through land use, land-use change, and forestry (LULUCF) account for about 62% of the total GHG emissions in Brazil^43^. In addition, LULUCF is a key sector for adaptation to climate change impacts. A major variable in the carbon emission estimate is the biomass content of the natural vegetation. However, the financial cost to systematically acquire *in situ* carbon estimates over large and structurally complex biomes, such as the Amazon, and the coarse resolution of remote sensing maps add layers of uncertainty to biomass maps. Airborne Laser Scanning (ALS) has evolved to provide an important contribution to the scientific debate and improve forest biomass representation.

The current map enables more detailed characterization of the forest structure, allowing for estimation of biomass within a wider range of values than the maps previously used in National Inventories. Soon, extensive verification will be possible using data from recently launched and upcoming orbital sensors, such as NASA’s GEDI or Earth Explorer Biomass from the European Space Agency.

## Data Availability

The code used to develop this work can be accessed through the following link: - Code for data cleaning and analysis is provided as part of the replication package. It is available at: https://zenodo.org/badge/latestdoi/93561048
